# Reversed double-beam photoacoustic spectroscopic analysis of photoinduced change in absorption of cellulose fibres

**DOI:** 10.1038/s41598-022-18749-w

**Published:** 2022-08-25

**Authors:** Levente Csóka, Worakan Hosakun, Ottó Kolonics, Bunsho Ohtani

**Affiliations:** 1 Faculty of Informatics, ELTE Eötvös Loránd University, 1053 Budapest, Hungary; 2 Institute of Cellulose and Paper Technology, Celltech-Paper Ltd., 9634 Lócs, Hungary; 3 Institute for Catalysis, Hokkaido University, Sapporo, 001-0021 Japan

**Keywords:** Biomaterials, Electrical and electronic engineering

## Abstract

Photoabsorption properties of cellulose fibres under continuous and modulated irradiation were investigated in situ by the use of reversed double-beam photoacoustic spectroscopy (RDB-PAS). This photoacoustic (PA) measurement enabled observation of ultraviolet- and visible light-induced, electron trap filling, and reductive change on the surface of the fibres. Energy-resolved measurements and analysis of the kinetics of photoinduced de-excitation suggested that electrons that accumulated in the different cellulose crystalline phases had moderate reactivity toward molecular oxygen. Saturation limits of the intensities of the PA and RDB-PAS signals under de-aerated conditions in the presence of surface-adsorbed methanol were estimated for softwood and hardwood cellulose samples. The results suggest that the RDB-PAS technique is a feasible method for the estimation of the electron trap distribution, which is a potential measure of the density of crystalline cellulose defects.

## Introduction

Cellulose is a naturally abundant polymer of linear β-1-4-linked d-glucose units. It can be found in lower and higher plants, marine animals, algae, valonia, fungi, bacteria, invertebrates and even amoebae. Fibrous cellulose is a water-insoluble substance that has a role in the maintenance of the structure of the cell walls in plants or marine animals.

This fascinating biopolymer was first discovered and isolated by Payen in 1838^1^. The chemical structure and physical properties of cellulose make it ideal for green and sustainable development. Herein, we summarize the basic features of it, which are related to the electrical properties. The physical properties of cellulose, for example interaction with electromagnetic radiation, are influenced by its supramolecular structure, namely the hydrogen-bonding network. Three kinds of hydroxyl groups bond equatorially in glucose residue under different polarities, and the resulting inter- and intrachain interactions are responsible for the stabilisation, crystalline allomorph structures and hydrophilic nature of cellulose. The hydrogen atom (H) of O3 in the reducing glucose ring serve as hydrogen-bond donator to O5 on the non-reducing ring, while the hydrogen of O2 oxygen atom (O) on the non-reducing ring donates to the O6 of the reducing ring, contribute as hydrogen-bond acceptor in the hydrogen-bonding network. In nature, cellulose chains are not stable individually, but gather into clusters that form fibrillary or fibre-like material. Pure cellulose subfibrils exist only in bacterial cellulose and valonia. Most of the higher-plant cellulose fibres are surrounded by hemicelluloses and lignin matrices.

Chemical pulping under high temperatures can be applied to remove hemicelluloses and lignin to isolate cellulose fibres. Imperfections occur in the geometrical arrangement of the Hs or Os in cellulose crystalline allomorphs; these are caused by temperature relaxation or mechanical deformations that occur during grinding. These crystal defects are called Frenkel- or Schottky-type voids, in which an ion is displaced or shifted to a nearby space in the lattice of solid dielectrics^2^. Imperfections can be responsible for ionic conduction, but at the same time can cause conduction to cease as they trap electrons. This trapping process means that materials such as cellulose can be effective insulators because the few conduction electrons are trapped at defects, so the stable environment for conduction electrons is eliminated.

Approximately 18 linear, β-1-4-linked glucan chains form subfibrils, which consist of repeating ordered (crystalline) and disordered (amorphous or chain-dislocating) sections in fibrils. Moreover, solution state cellulose exists, which has different structure and properties^3^. In the ordered cellulose-chain regions, strong and complex inter- and intramolecular hydrogen-bond networks are formed. Hence, cellulose subfibrils have different crystalline polymorphs. In native cellulose (cellulose I), which is found in nature, there are two intrachain hydrogen bonds, one in the O3–H···O5 and the other in the O2–H··· Ο6 sections, and one interchain hydrogen bond, which is in the O6–H···O3 section. The latter links the layers laterally and depends on the hydroxymethyl conformation at the C-6 position^4^. Native cellulose I consists of two distinct crystal phases: the Iα, a triclinic crystalline unit cell^5^ and the Iβ, a monoclinic crystalline unit cell^6^. The Ια/Ιβ ratio defines the properties of the cellulose. It varies within the cellulose microfibrils and among different cellulose sources. Algae and bacterial cellulose specimens are rich in the Iα form, while cotton, wood and ramie fibres are rich in the Iβ crystalline allomorph^7^.

Cellulose has a moderate, complex dielectric constant of approximately 5 and a permanent dielectric constant of approximately 4400^8^. In general, wood has weak electrical properties^9^, while cellulose nanocrystalline thin film has a high piezoelectric response of 2.1 Å V^−1^^10^, low ionic conductivity and surface charge capacity (8.24 × 10^−7^ ∆mAh) and high resistivity (4.2 × 10^13^ Ω)^11^.

The photoacoustic (PA) effect was first observed by Bell^12^ when he irradiated a sample with modulated sunlight. PA spectroscopy can be used to study the light induced non-radiative recombination of electron–hole pairs in semiconductors^13^ in order to evaluate thermal diffusivity^14^ during the de-excitation processes that occur in a sample. The technique can also be used to determine the band-gap of semiconducting materials^15–17^ as well as the numbers of impurities and positive holes, and the rate of defect-related absorption. In this study, we investigated the photoabsorption properties of cellulose fibre samples by use of modified PA spectroscopy (reversed double-beam PA spectroscopy, RDB-PAS) to identify the energy-resolved distribution of electron traps as a fingerprint.

## Materials and methods

Two different types of cellulose samples were used. Sample A was a total-chlorine-free (TCF), bleached, sulphate (kraft) softwood pulp sample from Södra Pulp Mill (type: blue Z, Sweden). Sample B was an elemental-chlorine-free (ECF), bleached, sulphate (kraft) hardwood pulp sample from Kotlas Pulp Mill (Koryazhma, Arkhangelsk Oblast, Russia). Specific surface areas were taken from the literature based on nitrogen adsorption and calculated based on the Brunauer–Emmett–Teller (BET) equation for softwood and hardwood pulp. The BET surface areas were ~ 1.8 and ~ 2 m^2^ g^−1^, respectively^18,19^.

PA and RDB-PAS measurements were performed as explained in a report^20^ but with some modifications. It is taken as a principle that, during the PA spectroscopic measurement, only the absorbed photons can generate an acoustic wave, while light-scattering or other optical properties do not affect the spectrum. A homemade PA cell was used; this consisted of an aluminium body (inside volume ≈ 0.5 cm^3^), a micro-electromechanical system (MEMS; SparkFun MEMS Microphone Breakout, INMP401 (ADMP401)) microphone module, and a quartz-glass window that was transparent over the 250–1000 nm range of measurements. The cell was placed in a sealed acrylic-resin box to minimise vibration noise and changes in temperature during the measurement. A cellulose pulp sample was placed in the cell, and PA spectra were measured at room temperature under a nitrogen atmosphere. Monochromatic light (≈ 0.2 mW cm^−2^) that was supplied by a monochromator (Bunkokeiki) equipped with a xenon lamp was modulated by a light chopper at 80 Hz. The PA signal that was acquired by the MEMS microphone module buried in the cell was amplified and monitored by a digital lock-in amplifier (NF LI5640). The PA signal was normalised through the use of carbon black powder as a reference to compensate for wavelength-dependent light intensity.

**Figure 1:**
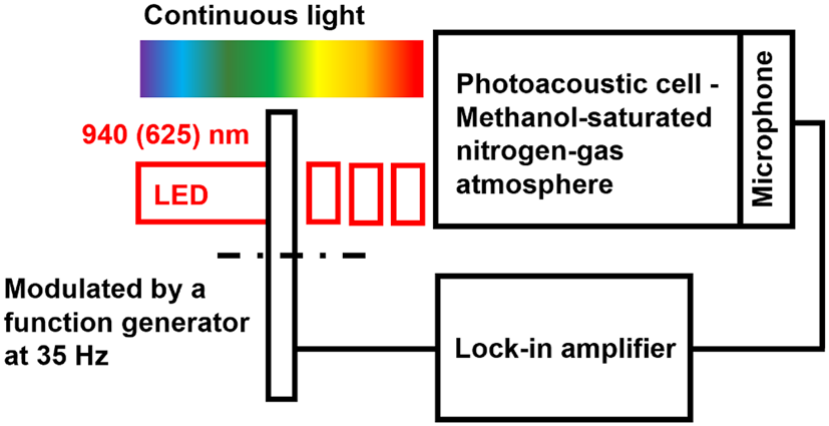
Schematic representation of the RDB-PAS set-up.

In addition to this ordinary, single-beam PAS measurement, RDB-PAS measurements were also carried out. These were performed with the use of wavelength-scanned (600–250 nm) monochromatic light along with a 35 Hz-modulated red (625 nm) light-emitting diode (LED) (Intelligent LED Solutions, RILI-ON01-RED1-SC211) or a near-infrared (940 nm) LED (Intelligent LED Solutions ILI-IO01-94SL-SC201). The set-up is shown in Fig. 1. The RDB-PAS signal was not normalised since the signal intensity, which corresponded to the total accumulated trapped-electron density, was independent of the continuous light intensity. The atmosphere in the cell was air for the PAS measurements and static methanol-saturated nitrogen for the RDB-PAS measurements. Before the measurement, the nitrogen gas was bubbling through methanol for 20–23 min at 30 ml/min flow.

## Experimental set-up

Figure 1 shows the concept by which RDB-PAS measurements are made, with details of the measuring apparatus and principles of the modulated PA signal generation. The PA signal was recorded in lock-in mode and averaged at each wavelength of continuous light during scanning from 620–250 nm in 5 nm steps. In RDB-PAS, the modulated- and continuous light beams (originally in DB-PAS mode) are exchanged. In this experiment, the continuous wavelength-scanning light was illuminated to excite electrons to enter ETs, and the given frequency of the modulated (chopped) monochromatic light was irradiated simultaneously with the cellulose sample to detect photoabsorption of electron-filled ETs. This photoinduced, trap-filling spectrum was recorded. Before the measurement, nitrogen gas that was saturated with methanol vapour was made to flow through the cell for to capture the positive holes irreversibly and to avoid the disappearance of once-trapped electrons through their reactions with positive holes.

## Results and discussion

**Figure 2:**
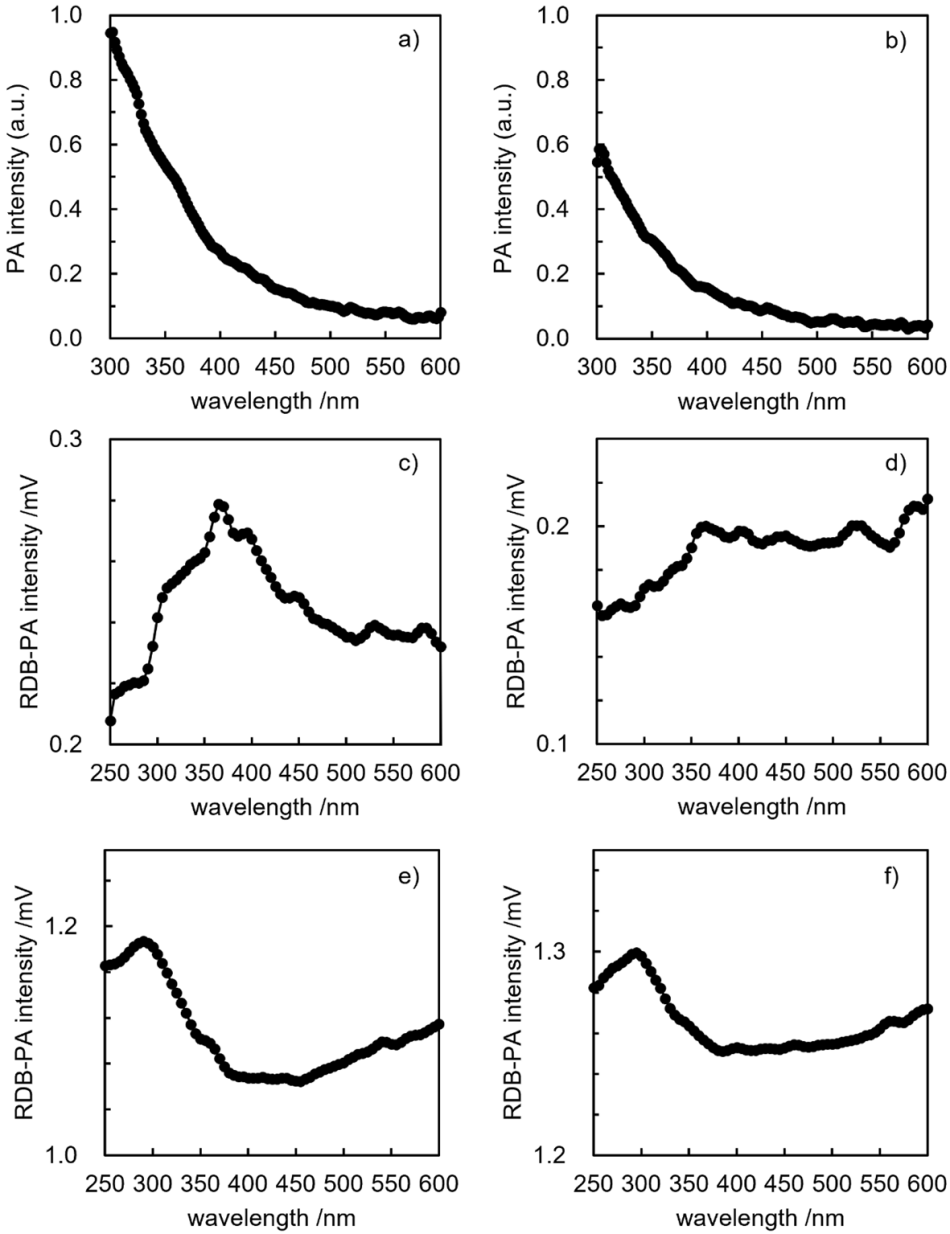
PA spectra of sample A—softwood pulp—(**a**) and sample B—hardwood pulp—(**b**); RDB-PA spectra with low-intensity 625 nm LED shown for sample A—softwood pulp—(**c**) and sample B—hardwood pulp—(**d**); and RDB-PA spectra with high-intensity 940 nm LED shown for sample A—softwood pulp—(**e**) and sample B—hardwood pulp— (**f**).

Due to the absorption of continuous and modulated light inside the sample, non-radiative processes and de-excitation occurred and caused heat generation. The heat that was periodically generated by the modulated light, not by the continuous light, was transferred to the gas in the cell due to thermal conduction and caused pressure oscillations at the modulation frequency. This oscillation was detected by the acoustic transducer (microphone) with the lock-in amplifier. The amplitude (weighted average) of the PA signals (Fig. 2a,b) depended on the amount of generated heat, which was strongly correlated with the optical absorption coefficient. By scanning the wavelength of modulated light from longer wavelengths to shorter wavelengths (without the LED and independently of the RDB-PAS measurement), the PA signal intensity (photoabsorption) of the samples A and B at 500 nm was first gradually and then steeply increased and was saturated at approximately 300 nm (Fig. 2a,b; sample saturation was greater for sample A than for sample B). A similar pattern can be found in the literature for microcrystalline cellulose, hemicelluloses and lignin^21^. As shown in the PA spectra (Fig. 2a,b), absorption of visible light by the two different types of cellulose was negligible and was of even lower intensity for the hardwood cellulose than for the softwood. This indicated that the optical absorption coefficient of ETs, even if it existed, was negligibly low due to the very low density of ETs. Consequently, it was practically impossible to measure the optical absorption of the valence band—ET by application of conventional photoabsorption spectroscopy in the case of a dielectric material. However, since the optical absorption coefficient of the valence band—ET was not zero, it was considered that irradiation over a prolonged period in the presence of a strong electron donor would enable accumulation of electrons in ETs and therefore the collection of measurable data. In the single-beam PAS measurement, the irradiation period of scanned modulated light was short (ca. 15 s), which was not long enough to enable electron filling, and the PA signal corresponded to the absorption of a given wavelength, not to the wavelength for ET absorption. In comparison, the irradiation period of the scanned modulated light in RDB-PAS was ca. 180 s. The intensity of the PA signal, which was related to heat generation inside the sample, was immeasurable when light absorption inside the sample was very weak, and the detection limit was determined by the noise signals that were produced by PA cell walls, the transmission window and various contaminants.

Heat generation in cellulose samples is low, a few W g^−1^ at room temperature^22^ and the optical absorption coefficient is weak because phonons must be generated to fulfil the requirements of momentum conservation during light absorption. This is an important advantage of PA spectroscopy over regular absorption measurements (combined transmission and reflectance measurements). If the latter is used, the determination of the absorption edge is difficult and particular conditions are optimal and/or the incident light is scattered due to sample surface irregularities, since only the absorbed light fraction causes the signal generation. The PA signal is proportional to the optical absorption coefficient until saturation occurs in the high absorption region. Therefore, in this experiment, measurements of PA signal intensity as a function of incident photon energy enabled us to determine the absorption edge. The absorption edge for sample A was 390.9 nm, which is equivalent to 3.172 eV; for sample B it was 389.1 nm, equivalent to 3.187 eV. These figures indicated that electrons had accumulated in ETs from the deeper (lower energy) side to the shallower (higher energy) side, resulting in photoabsorption in the visible region of the spectrum (400–600 nm). This was observed in the electron-accumulated cellulose fibre samples that were analysed through use of RDB-PAS (Fig. 2c,d).

Overall, methanol oxidation of positive holes liberated by photo-excitation may have proceeded slowly under visible light irradiation. In other words, highly efficient electron transfer may have scavenged almost all the electrons that were in ETs. This hypothesis suggests that those ETs were located predominantly on the surface of the cellulose fibres.

Since cellulose is a dielectric insulator, no ET transition can be caused by intensity-modulated light in the wavelength region of ca. 400–600 nm. Hence a 625 nm red LED was chosen in the first experiments. When the depth of the sample holder, i.e., the sample thickness, was reduced from the standard 1 mm to less than 0.5 mm, the saturation of PA signal intensity at ca. 3.5 eV was appreciably reduced. This showed that the upper 0.5 mm part of the sample contributed to the PA signal and therefore a sample thickness of 1 mm may be sufficient to obtain reproducible results.

The RDB-PAS spectra that were obtained through the use of the low-intensity 625 nm LED, and the high-intensity 940 nm LED are shown in Fig. 2c–f, respectively. Saturation of the RDB-PAS signal using the low-intensity LED at around 3.4 eV (360 nm) and using the high-intensity LED at around 4.1 eV (300 nm) was commonly observed for all the cellulose samples that were used in this study. Total accumulated ET density was plotted as a function of the photon energy of continuous light (*E*/eV = 1240/(wavelength/nm)) and was differentiated from the lower energy side to discover the energy-resolved distribution of ETs (ERDT) as a function of energy.

This finding suggested that, under appropriate measurement conditions and with sufficient waiting time (180 s), all the ETs in this insulator material were filled with electrons when it was irradiated with UV and there was a weak accumulation of electrons on the surface of the cellulose fibres when it was irradiated with visible light.

**Figure 3:**
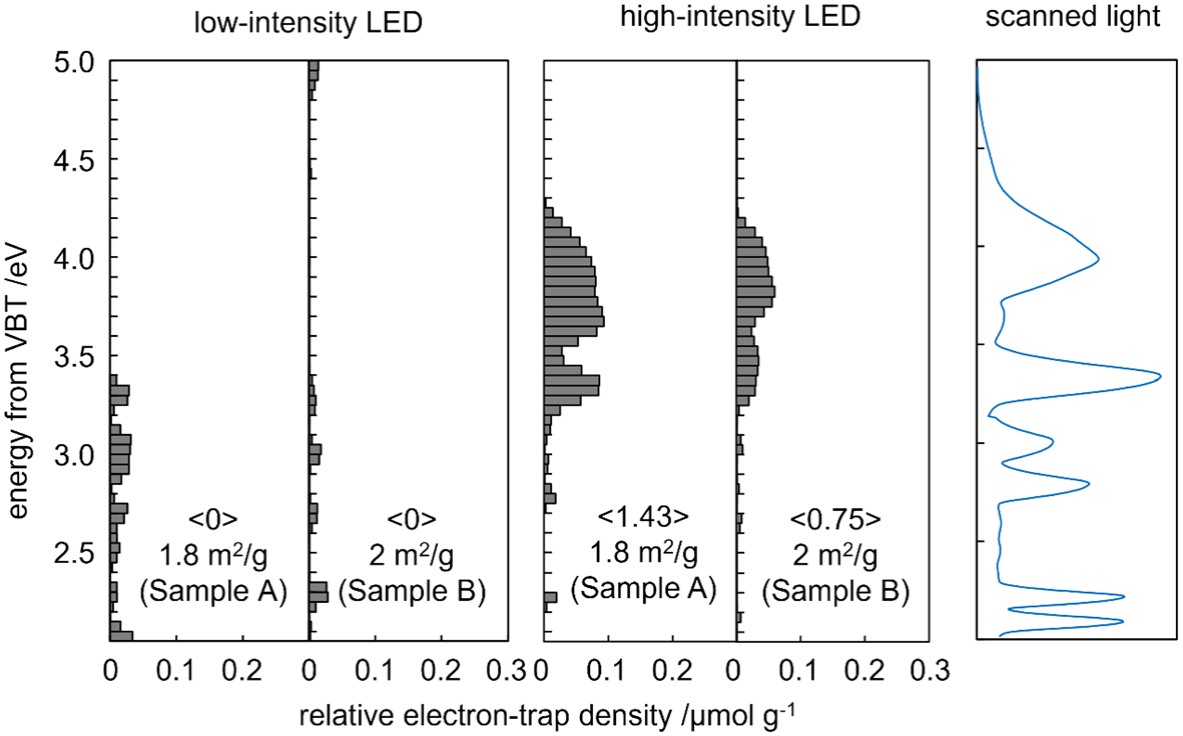
Energy-resolved distribution of electron traps in samples A and B of cellulose fibres under scanned, continuous light.

Since the RDB-PA spectra corresponded to the integrated form of the energy distribution of the ETs, the spectrum was differentiated from the lower energy side and an ERDT pattern was observed as shown in Fig. 3. The scale of absolute ET density was shown in the figures in μmol g^−1^ eV^−1^$;$ this scale was determined for titanium(IV) oxide (titania) samples, so for cellulose samples, this represented just relative values.

The two ERDT plots that are shown on the left in Fig. 3 are derived from the findings of the experiments with the low-intensity 625 nm LED source and can be interpreted as showing electron accumulation under visible light irradiation only. The third and fourth ERDT plots were generated from the findings of the studies performed with the high-intensity 940 nm LED light and they show appreciable results in the UV–Visible irradiation zone. It can be said that the low-intensity 625 nm LED experiment produced relatively large errors and was less informative than that performed with the high-intensity LED, which produced small but appreciable ERDTs that could be separated from noise. These results showed a few peaks at ca. 3.2–4.2 eV. Although the emission spectrum of the scanned excitation light (Fig. 3, right) showed emission peaks similar to those in the ERDT pattern, the observed ERDT peak positions might be affected by the intensity of the excitation light. The finding that the noticeable ERDT peaks appeared only when the high-intensity 940 nm LED was used suggests that ETs (or certain vacant electronic states) existed in the cellulose samples.

Both cellulose samples exhibited similar ERDT spectra when RDB-PAS results were compared. The patterns seemed to be structured in two main zones, which showed slight intensity variation between them, as described earlier. The shapes of the ERDT spectra were similar for the softwood and hardwood cellulose fibre samples. This finding indicated that the energy resolution in RDB-PAS was much higher than in other methods. Since the absolute molar amount (or density, in μmol g^−1^) could be determined through use of photochemical methods, the above-mentioned similarity in the pattern shapes suggested that PA signal intensity was proportional to the density of the ETs and that the optical absorption coefficient of electron-filled ETs at 625 nm was constant regardless of their energy levels. So far, no experimental results have been reported for photoabsorption spectra of electron-filled ETs according to their energy levels in cellulose samples. In the present RDB-PAS study, the use of wavelength-scanned modulated light rather than constant wavelength LED light may have enabled the measurement of the spectra of electron-filled ETs with different ET energies. In other words, the use of RDB-PAS produced ERDT spectra that showed relative ET densities. In Fig. 3, the figures in brackets (〈 〉) show the total ET densities. The figures below those show the specific surface areas of the cellulose fibres. As reported previously^23^, total ET density depends predominantly on the specific surface area of samples (those for cellulose fibres are two orders of magnitude lower than those for TiO_2_ semiconductor material) and on the main crystalline phase, which is identical for softwood and hardwood samples^24^. Therefore, these two parameters reflect the size of the bulk (surface) and the bulk structure, respectively. On the other hand, even if samples A and B have similar specific surface areas and the same crystalline phase, ERDT spectra are sometimes dissimilar. Differences in ERDTs are attributable to the surface structure and are not reflected in the total ET density. They may be affected by all the parameters that characterise cellulose materials: surface structure, bulk structure, bulk (surface) size, and cell differences that occur after digestion temperatures are relaxed.

## Conclusion

It has been shown that PA spectroscopy is a powerful tool for the study of the density of ETs in cellulose fibres. An important advantage of this method over regular absorption measurements is that it can be used to determine the ETs that are formed on the surface of cellulose fibres during temperature relaxation. PA spectroscopy can be applied to very thin layers of cellulose pulp, in which light absorption is weak and hard to detect, and for samples that scatter incident light. The RDB-PAS signal was recorded due to the electron trap filling on different cellulose fiber surfaces. In addition, it has been shown that the energy differences between the ETs are smaller in hardwood than softwood, because hardwood has fewer fibres but more vessels and other tissue elements than softwood, but the same crystalline phases and specific surface areas. The results reveal that cellulose possess a unique response to photo induced changes and can inspire a new approach of smart material design and promotes further applications.

## Data Availability

The datasets used and/or analysed during the current study available from the corresponding author on reasonable request.

## References

[1] Payen A (1838). Mémoire sur la composition du tissu propre des plantes et du ligneux (Memoir on the composition of the tissue of plants and of woody). Comptes Rendus.

[2] Murphy EJ (1974). High field conduction in native cellulose and its structural implications. J. Colloid Interface Sci..

[3] Ono Y, Furihata K, Isobe N, Saito T, Isogai A (2018). Solution-state structures of the cellulose model pullulan in lithium chloride/N,N-dimethylacetamide. Int. J. Biol. Macromol..

[4] Kondo T, Dumitriu S (2004). Hydrogen bonds in cellulose and cellulose derivatives. Polysaccharides.

[5] Nishiyama Y, Sugiyama J, Chanzy H, Langan P (2003). Crystal structure and hydrogen bonding system in cellulose Iα from synchrotron X-ray and neutron fiber diffraction. J. Am. Chem. Soc..

[6] Nishiyama Y, Langan P, Chanzy H (2002). Crystal structure and hydrogen-bonding system in cellulose Iβ from synchrotron X-ray and neutron fiber diffraction. J. Am. Chem. Soc..

[7] Habibi Y, Lucia LA, Rojas OJ (2010). Cellulose nanocrystals: Chemistry, self-assembly, and applications. Chem. Rev..

[8] Frka-Petesic B, Jean B, Heux L (2014). First experimental evidence of a giant permanent electric-dipole moment in cellulose nanocrystals. EPL.

[9] Bazhenov V (1965). Piezoelectric properties of wood. For. Chronicle.

[10] Csoka L (2012). Piezoelectric effect of cellulose nanocrystals thin films. ACS Macro Lett..

[11] Agarwal C, Singh MN, Sharma RK, Sagdeo A, Csóka L (2019). In situ green synthesis and functionalization of reduced graphene oxide on cellulose fibers by *Cannabis sativa* L. extract. Mater. Perf. Charact..

[12] Bell AG (1880). On the production and reproduction of sound by light. Am. J. Sci..

[13] Yamamoto H, Suemune I, Yamanishi M (1986). Photoacoustic study of surface and bulk nonradiative recombinations in GaAs with two-wavelength excitations. J. Appl. Phys..

[14] Jeyadheepan K, Palanichamy P, Swaminathan V, Jayachandran M, Sanjeeviraja C (2010). Thermal and optical properties of Cd2SnO4 thin films using photoacoustic spectroscopy. Appl. Phys. A Mater. Sci. Process..

[15] Zelaya-Angel O, Alvarado-Gil JJ, Lozada-Morales R, Vargas H, Ferreira Da Silva A (1994). Band-gap shift in CdS semiconductor by photoacoustic spectroscopy: Evidence of a cubic to hexagonal lattice transition. Appl. Phys. Lett..

[16] Astrath NGC (2006). Band gap energy determination by photoacoustic spectroscopy under continuous light excitation. Appl. Phys. Lett..

[17] Prías-Barragán JJ (2006). Band gap energy determination by photoacoustic absorption and optical analysis of Cd1-xZnxTe for low zinc concentrations. J. Crystal Growth.

[18] Bismarck A (2002). Surface characterization of flax, hemp and cellulose fibers: Surface properties and the water uptake behavior. Polym. Compos..

[19] Tang X (2021). Facile preparation of all-cellulose composites from softwood, hardwood, and agricultural straw cellulose by a simple route of partial dissolution. Carbohyd. Polym..

[20] Nitta A, Takashima M, Takase M, Ohtani B (2019). Identification and characterization of titania photocatalyst powders using their energy-resolved distribution of electron traps as a fingerprint. Catal. Today.

[21] Gould JM (1982). Characterization of lignin in situ by photoacoustic spectroscopy. Plant Physiol..

[22] Rac-Rumijowska O, Maliszewska I, Fiedot-Toboła M, Karbownik I, Teterycz H (2019). Multifunctional nanocomposite cellulose fibers doped in situ with silver nanoparticles. Polymers.

[23] Nitta A, Takase M, Takashima M, Murakami N, Ohtani B (2016). A fingerprint of metal-oxide powders: Energy-resolved distribution of electron traps. Chem. Commun..

[24] Chen Y (2017). Comparative characteristics of TEMPO-oxidized cellulose nanofibers and resulting nanopapers from bamboo, softwood, and hardwood pulps. Cellulose.

